# Effects of Lactic Acid Bacteria on Microbial Metabolic Functions of Paper Mulberry Silage: A BIOLOG ECO Microplates Approach

**DOI:** 10.3389/fmicb.2021.689174

**Published:** 2021-06-25

**Authors:** Xuekai Wang, Xinxin Cao, Han Liu, Linna Guo, Yanli Lin, Xiaojing Liu, Yi Xiong, Kuikui Ni, Fuyu Yang

**Affiliations:** ^1^College of Grassland Science and Technology, China Agricultural University, Beijing, China; ^2^National Engineering Laboratory for Tree Breeding, College of Biological Sciences and Biotechnology, Beijing Forestry University, Beijing, China; ^3^Beijing Sure Academy of Biosciences Co., Ltd., Beijing, China; ^4^Department of Animal and Food Sciences, University of Delaware, Newark, DE, United States

**Keywords:** paper mulberry, silage, lactic acid bacteria, additives, BIOLOG ECO, microbial communities, metabolic function

## Abstract

Lactic acid bacteria occupy an important position in silage microorganisms, and the effects of exogenous lactic acid bacteria on silage quality have been widely studied. Microbial metabolism has been proved as an indicator of substrate utilization by microorganisms. Paper mulberry is rich in free carbohydrate, amino acids, and other components, with the potential to be decomposed and utilized. In this study, changes in the microbial metabolism characteristics of paper mulberry silage with *Lactiplantibacillus plantarum* (LP) and *Lentilactobacillus buchneri* (LB) were studied along with a control (CK) using BIOLOG ECO microplates. The results showed that average well-color development (AWCD), Shannon diversity, Shannon evenness, and Simpson diversity exhibited significant temporal trends. LB and LP responded differently in the early ensiling phase, and the AWCD of LB was higher than LP at 7 days. Principal component analysis revealed that CK, LB, and LP samples initially clustered at 3 days and then moved into another similar cluster after 15 days. Overall, the microplates methodology applied in this study offers important advantages, not least in terms of accuracy.

## Introduction

Paper mulberry (*Broussonetia papyrifera* L.) is rich in crude protein and widely adaptable in temperate, subtropical, and tropical regions. It has been recently developed as a new type of fodder (Bingwen et al., 2019; Du et al., 2020). As an important technique for preserving forage nutrients in the context of seasonal harvests in climates characterized by high temperatures and plentiful precipitation, ensiling has been proved to be an easy way to preserve paper mulberry (Zhang et al., 2019). In the process of ensiling, chemical components are either maintained or partially converted into distinct substances by microorganisms. In addition to carbohydrates, paper mulberry also contains abundant amino acids (Wang et al., 2019) and many other bioactive compounds (Ming et al., 2011; Cao et al., 2020). At the beginning of silage fermentation, undesirable microbial compositions containing yeast, mold, and enteric bacteria are commonly detected, which can directly and indirectly affect silage safety (Muck, 2010). Exogenous lactic acid bacteria are often employed to accelerate the procedure, inhibit the growth of undesirable microorganisms, and improve the quality of silage. However, little is known about the metabolic characteristics of microbial communities in paper mulberry silage, which significantly limits the optimization of the processing technology of silage fermentation.

The plate colony counting method (Kramer et al., 1979), nucleic acid amplification technique (Zhao and Dong, 2012), and cell morphology (Smith et al., 2016) are conventional approaches for evaluating the structure and composition of microbial communities. However, considering the complicated operation and assays and low repeatability, the overall status of microbial communities is difficult to describe quickly, conveniently, and comprehensively by these above methods. Thus, it is highly desirable to develop a new method for assessing the status of microbial communities. The BIOLOG ECO microplate is a technique for characterizing microbial communities derived from samples based on metabolic functions. Profiles of metabolic functions are assessed using a redox system with 31 different sole carbon sources and one blank control (water), repeated three times (96 wells in total) in each microplate (Gryta et al., 2014). In these plastic microtiter plates, the grade of substrate utilization by microorganisms is quantified based on the absorbance of each well. Different carbon sources, which represent particular metabolic functions, can be used to compare microbial communities in different samples, indicating that the BIOLOG ECO microplate methodology has the potential to measure and analyze the status of microbial communities (Choi and Dobbs, 1999).

Due to the biological and biochemical properties of BIOLOG ECO microplate, it is a relatively simple and quick method for describing the ecological diversity and community-level status of environmental microorganisms. Indeed, it has been widely used in various contexts, such as soils (Bradley et al., 2006), fertilizer (Wei et al., 2018), stored rice (Ge et al., 2018), sludge (Wang et al., 2018), and wastewater (Choi and Dobbs, 1999). However, its potential in the field of silage remains to be elucidated.

In view of the foregoing, this study aimed to investigate the metabolic functions in paper mulberry silage inoculated with *Lactiplantibacillus plantarum* (LP) and *Lentilactobacillus buchneri* (LB), based on an analysis of metabolic profiles, activity, and diversity. The BIOLOG ECO microplate technique was utilized to assess the effect of silage duration (from 0 to 60 days) on the characteristics of different microbial communities. The results of this study provide insights into the effects of ensiling and lactic acid bacteria additives on the metabolic function of microbial communities over time.

## Materials and Methods

### Silage and Samples Preparation

The LP was from woody forage silages while LB was from Sichuan pickles (Gaofuji, China). After isolation and purification, both strains were transformed to additives via lyophilization according to a previous study (Zhang et al., 2016). Paper mulberry was harvested at 120 cm and chopped into approximately 2 cm long pieces using a combine harvester. Fragments were separately exposed to different ensiling treatments with an equal amount of distilled water and divided into three groups, namely additives of LP, additives of LB, and the blank control (CK). The additives were given at a level of 10^6^ cfu per gram of raw material (RM) and mixed evenly. Every 3 kg of forage was compressed to a density of around 593 kg/m^3^ and sealed in a polyethylene bag (45 cm × 75 cm) by a vacuum sealing machine. For each treatment group, four replicates (one as backup) were prepared on each ensiling day. A total of 60 bags was stored in a dark room at room temperature (around 25°C). Silages collected at 0 day (raw material), 3, 7, 15, 30, and 60 days were applied to observe the changes in microbial metabolism. Forages were homogenized and sub-samples of about 100 g were separated by coning and quartering methods repeatedly. Next, 10 g was removed and dipped in 90 mL of sterilized saline solution (0.85% NaCl). The mixture was vortex-homogenized for 30 s and 1 h at 4°C, 150 rpm by a Controlled Crystal Oscillator (RONGHUA, China). To enrich the microorganisms, filtration was undertaken through a double-layer sterile gauze and centrifuged at 7,800 rpm for 15 min at 4°C. The pellet was resuspended and washed with the above solution twice, and finally made up to 100 ml. Each aliquot of well-mixed diluent (150 μL) was added into the wells of BIOLOG ECO microplates completely within 1 h. Once inoculated, the microplates were placed in a dark and anaerobic container at 25°C. During incubation, the absorbance values were recorded at a wavelength of 590 nm at an interval of 24 h for a total of 240 h using a microplate readers (TECAN, Swiss).

### BIOLOG ECO Analysis

The production of NADH via microbial respiration reduces tetrazolium dye to formazan, resulting in chroma development expressed as Average Well-Color Development (AWCD) (Garland and Mills, 1991). The wells of BIOLOG ECO microplates were filled with six carbon sources, namely carboxylic acids, carbohydrates, amino acids, polymers, and miscellaneous and amines/amides, which were used to measure the capability of microorganisms with different carbon sources in microbial communities (Ge et al., 2018). To detect the changes, it was necessary to study the differences between their metabolic characteristics with different carbon sources. Evaluation was conducted by principal component analysis (PCA) with transformed data (Kong et al., 2013). Absorbance value data were calculated as AWCD and standardized as *R*_*si*_ via the following equations:

(1) $$\text{AWCD}=\sum_{i=1}^{31}\left(C_{i}-R\right)/31$$

(2) $$R_{si}=\left(C_{i}-R\right)/\text{AWCD}$$

In each replicate, *C*_*i*_ represents the absorbance value of each reaction well at 590 nm, and *R* represents the absorbance value of the water blank well. Data of (*C*_*i*_ – *R*) less than 0.06 were regarded as zero (Classen et al., 2003). Data from the 144 h were used for the PCA.

### Microbial Diversity Analysis

The Shannon-Wiener diversity index (H’), Shannon evenness index (E), and Simpson diversity index (D) were used to investigate metabolic functional diversity at the community level (Hiraishi et al., 1991; Hu et al., 1999; Keylock, 2005).

(3) $$P_{i}=\left(C_{I}-R\right)/\sum \left(C_{i}-R\right)$$

(4) $$H^{\prime }=-\sum P_{i}\ln P_{i}$$

(5) $$E=H^{\prime }/\ln S$$

(6) $$D=1-\sum P_{i}^{2}$$

where *P*_*i*_ represents the ratio of the absorbance value in the *i*-th (1–31) well to the total absorbance value of all wells. *S* represents the number of utilized carbon sources among 31 carbon sources.

### Substrate Utilization Abundance

To visualize the utilization of various substrates in the BIOLOG ECO microplates by the microorganisms as the average absorbency proportion of each carbon source, the data were divided into six groups according to the type of carbon source based on the following equations:

(7) $$f_{i}=\frac{C_{i}}{\sum_{i=1}^{31}C_{i}}$$

(8) $$F_{i}=\frac{1}{n}\sum_{i=1}^{n_{j}}f_{i}$$

where *f*_*i*_ represents the OD fraction of carbon source species *i*, *F*_*j*_ represents the average OD fraction of the *j* kind of carbon source, and *nj* represents the number of wells in the replicate.

### Statistical Analysis

Using the JMP 14 software package (SAS Institute), the data were processed statistically and submitted to the PCA. Tukey’s test was used for multiple comparisons, with statistically significant differences at *p* < 0.05. All figures were generated with Origin 9.4.

## Results

### Microbial Metabolic Activity

**FIGURE 1:**
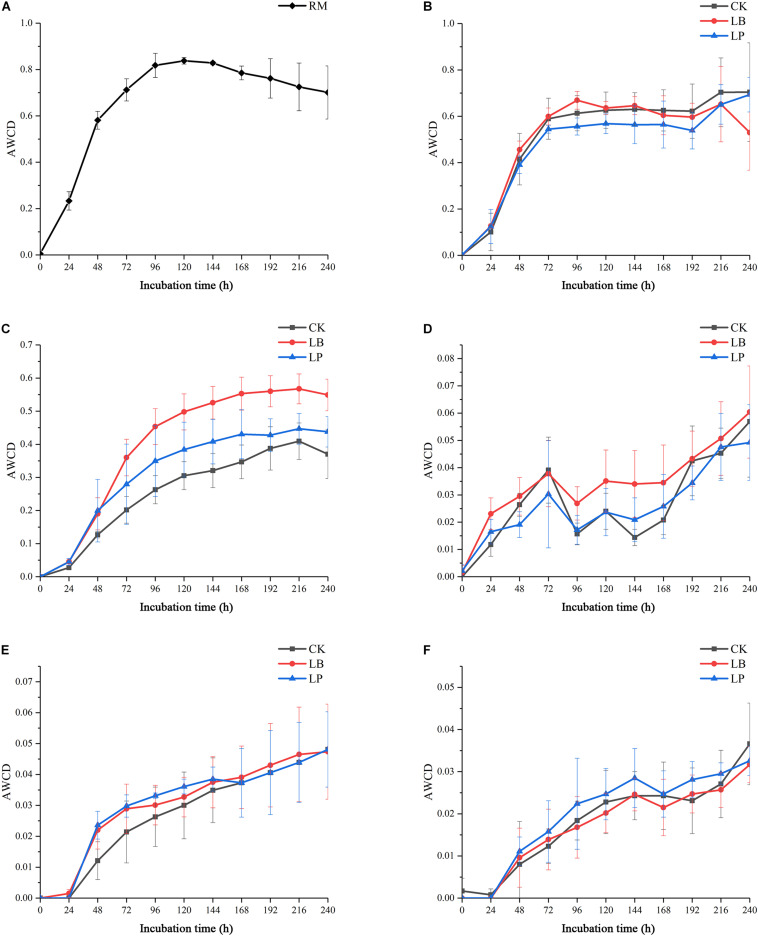
Average well-color development (AWCD) of BIOLOG ECO microplates within incubation time of microbial communities from paper mulberry RM (raw material) **(A)** and silages treated with blank control (CK), *Lentilactobacillus buchneri* (LB), and *Lactiplantibacillus plantarum* (LP) for 3 days **(B)**, 7 days **(C)**, 15 days **(D)**, 30 days **(E)**, and 60 days **(F)**.

To evaluate the metabolic activity of microbial communities of silages and raw materials, the development of AWCD of all carbon sources was investigated. As shown in Figure 1, all samples showed an increase in AWCD at the early stage, indicating that microbial communities from silage samples could metabolize carbon substrates in BIOLOG ECO microplates (Miyake et al., 2016). Then, the AWCD of the raw materials exceeded 0.8, reached an inflection at 144 h after incubation, and then began to decrease (Figure 1A); this accords with the findings of Zhou et al. (2019). A similar pattern was observed in the silages fermented at 3 and 7 days (Figures 1B,C), and the value was below 0.7 when the scatter plot forms a smooth curve. Furthermore, sample 7LB showed an evidently higher metabolic rate of the substrates than the other two inoculation stages, which indicated that the utilization of substrates by 7LB was most efficient. Compared with silage at the early stage, microbial communities of samples ensilaged for 15, 30, and 60 days displayed irregular growth paths (Figures 1D–F), and their AWCD curves were reduced by more than 10 times.

### Principal Component Analysis of Microbial Community

**FIGURE 2:**
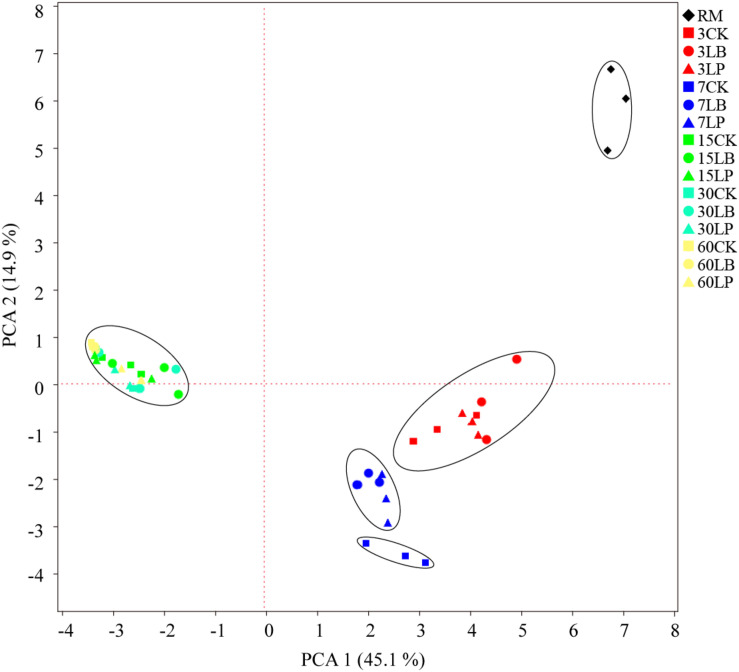
Principal component analysis (PCA) of microbial communities from paper mulberry RM (raw material) and silages treated with CK, LB, and LP for 3, 7, 15, 30, and 60 days after inoculation for 144 h.

**FIGURE 3:**
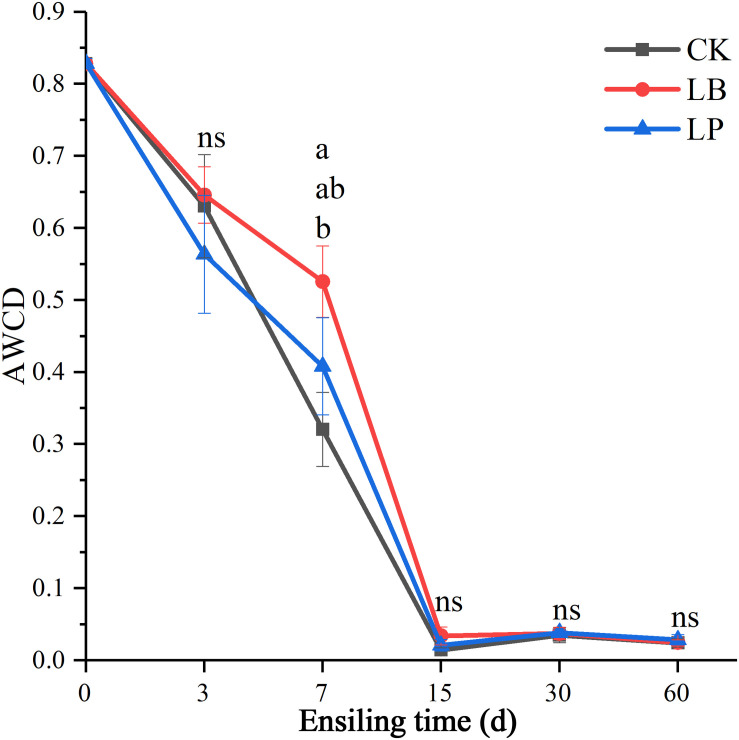
Average well-color development of all carbon sources of microbial communities from paper mulberry RM (raw material) and silages treated with CK, LB, and LP for 3, 7, 15, 30, and 60 days after inoculation for 144 h.

Figure 2 indicated the information of microbial communities with 31 carbon sources in the stable period of culture (at the time of inoculation 144 h). Principal component analysis (PCA) is a multivariate ordination method that is commonly used to analyze BIOLOG ECO data; it can differentiate the microbial communities of samples in a certain environment on different positions (Figure 2). Specifically, PC1 and PC2 accounted for 45.1 and 14.9% of the total variance, respectively. It is clearly discernable from Figure 2 that the samples were divided into five groups: (i) RM (raw material), (ii) ensiling for 3 days, (iii) LP and LB ensiling for 7 days, (iv) CK ensiling for 7 days, and (v) ensiling for 15, 30, and 60 days. Only the point position of RM group was obviously clustered in one sector and distinct from the other groups, suggesting that the microbial community was substantively different from silage samples. After ensiling for 3 days, no difference was observed between treated and untreated samples, while there was slight separation in ensiling at 7 days. Interestingly, the treated and untreated samples could not be well separated from each other after ensiling for 15 days. After inoculation of microbial communities for 144 h (Figure 3), no difference in AWCD values between the three additives was observed except ensiling for 7 days. Sample 7LP was much smaller than 7LB (around 0.41 and 0.53, respectively).

### Comparison of Metabolic Functional Diversity Indices

To further compare the metabolic functional diversity after different treatments, the Shannon diversity index (H’), Shannon evenness index (E), and Simpson index (D) after incubation for 144 h are shown in Table 1. Significant differences (*p* < 0.05) were found among the three indexes as a function of fermentation time. All of them exhibited analogous trends in decreasing quickly and then increasing slowly with the progression of silage time. An inflection point appeared 15 days after silage in the H’ index and D index and on 7 days in the E index. This indicates that the species richness and evenness of microbial communities in paper mulberry silage were high at the early stage of fermentation and tended to decrease and then rebounded slightly during ensiling. At the inflection points of each index, both additives LB and LP increased three indexes value, and LB was higher than LP. The H’ index and D index changed synchronously, indicating that species richness and the most common species in silage microbial communities in each treatment were closely related. With the extension of ensiling time, there was no longer a significant difference after 30 days (15 days) in the H’ index and D index of CK (LB and LP).

**TABLE 1:** Comparison of metabolic functional diversity indices of paper mulberry silage microbial communities.

Item	Ad	Ensiling time (*T*)					SEM	*p*-Value		
		3 days	7 days	15 days	30 days	60 days		*T*	Ad	T × Ad
H′	CK	2.888^a^	2.457^b^	1.333^d^	1.814^c^	1.776^c^	0.141	<0.001	<0.001	0.049
	LB	2.984^a^	2.717^a^	1.839^b^	1.933^b^	1.753^b^				
	LP	2.926^a^	2.597^a^	1.593^b^	1.861^b^	1.883^b^				
E	CK	0.940^b^	0.900^c^	0.962^ab^	0.984^a^	0.991^a^	0.006	<0.001	0.601	0.021
	LB	0.952^ab^	0.935^b^	0.955^ab^	0.975^a^	0.979^a^				
	LP	0.943^bc^	0.932^c^	0.968^ab^	0.983^a^	0.971^ab^				
D	CK	0.940^a^	0.899^b^	0.724^d^	0.832^c^	0.828^c^	0.017	<0.001	0.001	0.003
	LB	0.945^a^	0.928^a^	0.826^b^	0.848^b^	0.819^b^				
	LP	0.942^a^	0.918^a^	0.783^b^	0.840^b^	0.840^b^				

*H′, Shannon diversity; E, Shannon evenness; D, Simpson diversity; T, silage time; Ad, additives; SEM, standard error of the mean. ^a–d^Means in the same row with different superscripts differ significantly (p < 0.05).*

### Metabolic Utilization of Biochemical Categories Substrates

**FIGURE 4:**
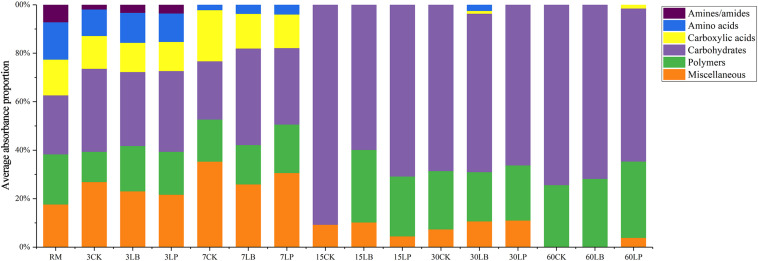
Substrates utilization profiles of microbial communities from paper mulberry RM and silages treated with CK, LB, and LP for 3, 7, 15, 30, and 60 days after inoculation for 144 h.

Substrate utilization profiles of the various carbon sources were classified and studied based on the average absorbance proportion of each carbon source (Figure 4). The results indicated that the substrate utilization profiles were different in the process of silage fermentation. In the RM, 3CK, 3LB, and 3LP samples, the six carbon sources were used to varying degrees. In the comparison of RM with ensiling for 3 days, the fractional content of carboxylic acids, amino acids, and amines/amides decreased slightly but not significantly, while the fractional content of carbohydrates increased from 24% to around 32%, suggesting that carbohydrates could be more easily utilized. With the increase in fractional content of carboxylic acids and miscellaneous for all ensiling for 7 days, the fractional content of carbohydrates for 7LB and polymers for 7CK and 7LP increased. The fractional content of miscellaneous ranked first (around 35%) in 7CK, while carbohydrates were used as the main fractional content in the 7LB (around 40%). Interestingly, in the 7LP, both miscellaneous and carbohydrates contributed almost equal contributions (around 31%). It is noteworthy that, during ensiling, the fractional content of amines/amides decreased for the first time on 7 days, and this trend occurred in carboxylic acids and amino acids on 15 days. In samples ensiled for more than 15 days, the fractional content of carbohydrates was predominant, followed by polymers (except 15CK).

**FIGURE 5:**
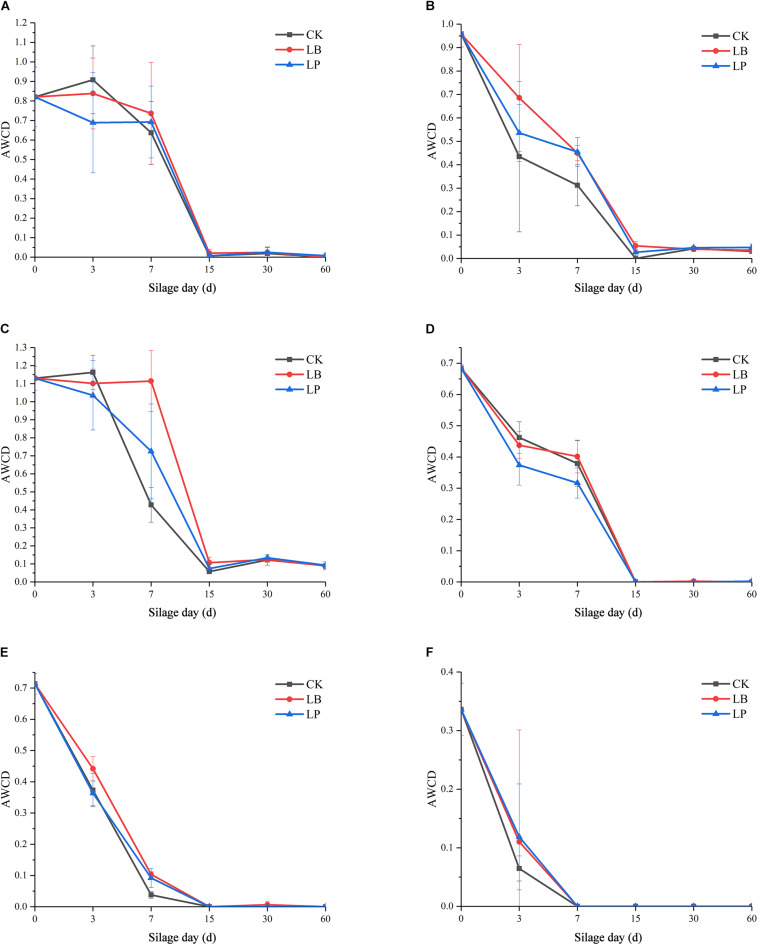
Average well-color development of six types of carbon sources microbial communities from paper mulberry RM and silages treated with CK, LB, and LP for 3, 7, 15, 30, and 60 days after inoculation for 144 h, including miscellaneous **(A)**, polymers **(B)**, carbohydrates **(C)**, carboxylic acids **(D)**, amino acids **(E)**, and amines/amides **(F)**.

Figure 5 shows the change of the AWCD associated with the six types of carbon sources as a function of ensiling time. At the beginning of ensiling, the AWCD of different substrates varied significantly. After 3 days, 3CK had the highest capability to utilize miscellaneous (a) and carbohydrates (c), while 3LP was the lowest content in both substrates. The biggest AWCD difference among the three treatments appeared in carbohydrates (c) in ensiling for 7 days, which indicated that the microbial communities of mulberry silage in the experiment caused high variations in carbohydrate metabolism with different additives. Moreover, the three treatments on carboxylic acids (d), amino acids (e), and amines/amides (f) had similar effects, with no significant difference during ensiling.

## Discussion

Microbial metabolism is regarded as a key link in the decomposition and transformation of nutrients in forages. Metabolism of undesirable microorganisms may lead to dry matter loss or a strong, pungent odor (Kung et al., 2018). Composed of a complex mixture of bacteria, yeasts, and molds, the microbiome associated with freshly harvested forage plays a key role in the ensiling process (Langston and Bouma, 1960). There are a variety of techniques available for assessing the differences in microbial communities during ensiling, including the plate counting method, denaturing gradient gel electrophoresis, single strand conformation polymorphisms, and other molecular approaches (Duniere et al., 2017). However, many of these classical approaches focus on characterizing the species, number, structure, and diversity of silage microbial communities but are limited in terms of metabolism. BIOLOG ECO microplate technology can competently describe metabolic functions of microbial communities, especially for environmental microorganisms (Choi and Dobbs, 1999). In this study, the microbial metabolic functions of microbial communities from paper mulberry silages were profiled with microbial metabolic activity and metabolic functional diversity indices via BIOLOG ECO microplate technology. The results suggested that this approach is feasible for studying silage microorganisms.

According to the major metabolic differences, especially the metabolism of carbohydrates, lactic acid bacteria are usually classified as obligate homofermentative, facultative heterofermentative, and obligate heterofermentative (Kandler, 1983). For making silage, LP is the most common bacterial inoculant, which was hitherto considered homofermentative but now is recognized as a taxonomically facultative heterofermentative strain (Pahlow et al., 2003). LB is the dominant species to improve aerobic stability in obligate heterofermentative additives (Kleinschmit and Jr, 2006). Due to the versatility of the BIOLOG system, it has the potential to distinguish the effects of LP and LB on metabolic activity of silage microbial communities. The AWCD decreased with fermentation time, implying that metabolic activity of silage microbial communities declined gradually. In addition, AWCD of microorganism samples from 7 days silages exhibited obviously different culture curves, listed in descending order as 7LB > 7LP > 7CK, which may be attributed to the improvement of metabolic capacity by the proliferation of lactic acid bacteria induced by additives (Siragusa et al., 2009). Homofermentative and heterofermentative additives could increase the number of lactic acid bacteria in the early stage of ensiling, and produce a large amount of lactic acid or acetic acid by metabolizing. Therefore, the low pH from lactic acid and acetic acid stabilizes silage fermentation by inhibiting the population growth and metabolic activity of spoilage microorganism intolerant of a low pH.

The substrate utilization profiles and PCA (Figure 2) were highly correlated. Samples separated in distinct clusters, suggesting differences in microbial community structures. This could have been caused by changes in the microbial communities of paper mulberry silage under low acidity conditions, which would be consistent with the findings of Zhang et al. (2019). As the accumulation of various organic acids increases, some species or strains reach a point where their metabolic activity drops off, which has no impact on the effectiveness (Cai et al., 2014). This was also confirmed by higher microbial metabolic activity at the early stage than that after 15 days (Figure 3).

Different metabolites of microbial communities could be regarded as the final display of different lactic acid bacteria additive (Xu D. et al., 2020). Similar to previous work on the bacterial diversity of alfalfa silages as determined by sequence technology (Guo et al., 2018), ensiling led to changes in microbial community diversity in paper mulberry. This was apparent in the dynamics of the Shannon index and Simpson index, with unambiguous heterogeneities between the CK group and silages treated with LB or LP. Interestingly, lactic acid bacteria additives have previously been reported to accelerate the convergence of the microbial community during ensiling (Xu S. et al., 2020), but the opposite result was found in our study, where the three treatments showed the same trends in terms of Shannon evenness.

In previous studies, regardless of enriching lactic acid bacteria in fresh raw materials (Chen et al., 2017) or adding external lactic acid bacteria artificially during ensiling (Zhang et al., 2020), relative abundance or cultivable quantity of various species of microorganisms have been the principal consideration rather than metabolic functions. Thus, there might be some neglected effects from additives or environmental factors in the silage fermentation process. The above results support this perspective.

## Conclusion

The present study focused on changes in microbial metabolic characteristics and functions of microbial communities in paper mulberry silages. Metabolic activities of microbial communities declined with ensiling time. After extension of the period to 15 days, the utilization of amino acids and carboxylic acids was stabilized at a low level. In addition, lactic acid bacteria additives gave evident signals on the effects of metabolic functional diversity indices. Both LB and LP accelerated the fermentation process in a steady state. Within this period, the metabolic functional diversity of LB was greater than that of LP.

By evaluating the functional diversity of silage microbial communities using the BIOLOG ECO microplates approach, this study provides insights into the effects of lactic acid bacteria additives on microbial communities’ metabolic functions, whilst concomitantly offering support for the increased diffusion and use of this methodology because of its advantages in terms of ease of operation and accuracy.

## Data Availability Statement

The original contributions presented in the study are included in the article/supplementary material, further inquiries can be directed to the corresponding author.

## Author Contributions

XW, XL, KN, and FY designed the study and wrote the manuscript. XW, XC, HL, and LG performed the experiments. XW, YL, and YX conducted the statistical analysis and visualization. KN and FY were involved in the revision of the manuscript. All authors reviewed and approved the final manuscript.

## Conflict of Interest

YL was employed by company Beijing Sure Academy of Biosciences Co., Ltd. The remaining authors declare that the research was conducted in the absence of any commercial or financial relationships that could be construed as a potential conflict of interest.
